# Goth migration induced changes in the matrilineal genetic structure of the central-east European population

**DOI:** 10.1038/s41598-019-43183-w

**Published:** 2019-05-01

**Authors:** I. Stolarek, L. Handschuh, A. Juras, W. Nowaczewska, H. Kóčka-Krenz, A. Michalowski, J. Piontek, P. Kozlowski, M. Figlerowicz

**Affiliations:** 1 Institute of Bioorganic Chemistry, Polish Academy of Sciences, Poznan, Poland; 2 Department of Human Evolutionary Biology, Institute of Anthropology, Faculty of Biology, Adam Mickiewicz University, Umultowska 89, Poznan, 61-614 Poland; 3 Department of Human Biology, Faculty of Biological Sciences, Wroclaw University, Wroclaw, Poland; 4 Institute of Archaeology, Adam Mickiewicz University, Poznan, Poland; 5 Institute of Computing Sciences, Poznan University of Technology, Poznan, Poland

**Keywords:** Biological anthropology, Population genetics

## Abstract

For years, the issues related to the origin of the Goths and their early migrations in the Iron Age have been a matter of hot debate among archaeologists. Unfortunately, the lack of new independent data has precluded the evaluation of the existing hypothesis. To overcome this problem, we initiated systematic studies of the populations inhabiting the contemporary territory of Poland during the Iron Age. Here, we present an analysis of mitochondrial DNA isolated from 27 individuals (collectively called the Mas-VBIA group) excavated from an Iron Age cemetery (dated to the 2^nd^-4^th^ century A.D.) attributed to Goths and located near Masłomęcz, eastern Poland. We found that Mas-VBIA has similar genetic diversity to present-day Asian populations and higher diversity than that of contemporary Europeans. Our studies revealed close genetic links between the Mas-VBIA and two other Iron Age populations from the Jutland peninsula and from Kowalewko, located in western Poland. We disclosed the genetic connection between the Mas-VBIA and ancient Pontic-Caspian steppe groups. Similar connections were absent in the chronologically earlier Kowalewko and Jutland peninsula populations. The collected results seem to be consistent with the historical narrative that assumed that the Goths originated in southern Scandinavia; then, at least part of the Goth population moved south through the territory of contemporary Poland towards the Black Sea region, where they mixed with local populations and formed the Chernyakhov culture. Finally, a fraction of the Chernyakhov population returned to the southeast region of present-day Poland and established the archaeological formation called the “Masłomęcz group”.

## Introduction

During the last decade, genetics and genomics have become new driving forces that have stimulated the rapid development of studies on the human past. Archaeogenomics has quickly evolved from analyses of single individuals to studies involving dozens of subjects^1–3^. As a result, an increasingly precise map of the genetic history of a human population is generated. Despite the progress in our understanding of the demographic processes that took place in Europe since its first peopling, the map still has a numerous blank spaces^4^. They are especially frequent in the case of the Early Bronze Age (EBA) and later periods, when more complex demographic and cultural events occurred^5–11^. Importantly, not all geographical regions have been sufficiently sampled^12^. The majority of data are confined to Central-West Europe, whereas Eastern Europe is scarcely represented^1^.

**Figure 1:**
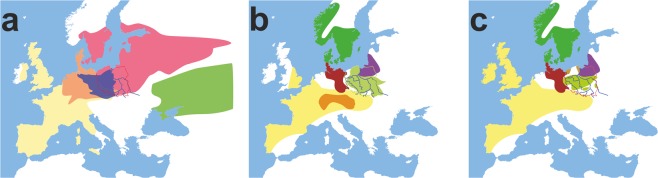
Geographical distribution of the archaeological cultures in Europe in the (**a)** LN/EBA: green, Yamnaya Culture 3300–2300 BC|pink, Corded Ware Culture 2900–2350 BC|yellow, Bell Beaker Culture 2900–1800 BC|orange, Corded Ware/Bell Beaker Culture|violet, Unetice Culture 2300–1600 BC; (**b)** BA/IA: green, Nordic Bronze Age 1700–500 BC|yellow, Hallstatt Culture 800–500 BC|orange, Hallstatt Culture Core|light green, Pomeranian Culture 700–300 BC|red, Jastorf Culture 600–100 BC|violet, Western Baltic Kurgans Culture 650–50BC; (**c)** IA prior to migrations period: green, Nordic Bronze Age 1700–500 BC|yellow, La Tene Culture 450–50 BC|light green, Przeworsk Culture 300 BC – 500 AD|red, Jastorf Culture 600–100 BC|violet, Western Baltic Kurgans Culture 650–50 BC|brown, Oksywie Culture 200 BC – 100 AD. Europe map by Roke, retrieved from https://commons.wikimedia.org/wiki/Category:Blank_maps_of_Europe#/media/File:BlankMap-Europe-v3.png, used under Creative Commons Attribution-ShareAlike 3.0 Unported (https://creativecommons.org/licenses/by-sa/3.0/deed.en), modified with Corel Draw ver. 12.0.

Recently, the first efforts have been undertaken to determine the genetic roots of peoples living in Central-East and Eastern Europe^13^. The works of Chylenski *et al*. and Lorkiewicz *et al*.^14,15^ suggest that the matrilineal genetic makeup of people living in the Vistula River Basin during the Early Neolithic (EN) and the Middle Neolithic (MN) better resembled the Funnel Beaker culture populations (TRB, ger. Trichter(-rand-)becherkultur), which were a mixture of Mesolithic Hunter Gatherers and Neolithic farmers, than the Linear Pottery culture-associated populations (LBK, ger. Linearbandkeramik), which were mostly composed of Neolithic farmers^13,16,17^. The studies of autosomal DNA of the Globular Amphora culture (GAC) population inhabiting the Vistula River Basin during the Late Neolithic (LN) showed no presence of Yamnaya steppe herder ancestry (YAM)^1,18^. At the same time, the Corded Ware culture (CWC) groups that later succeeded GAC have as much as 75% of their genetic makeup attributed to YAM (Fig. 1a)^7,17,19^. In particular, we know little about the genetic history of the populations that inhabited the territory of contemporary Poland in the Bronze Age (BA). During that time, cremation of the dead became a common custom; thus, our knowledge on these peoples is based almost exclusively on archaeological artifacts. They indicate that during the BA, several local cultures developed in this region^20^. All of them were, to a large extent, related to each other, and consequently, they are usually considered as one, called the Lusatian culture, existing from the EBA to the early Iron Age (IA). At the beginning of the IA (approximately 600 B.C.), an alternative to Lusatian culture, called the Pomeranian culture (PC), was developed. The PC dominated the region and spread between the Oder and Bug Rivers until the end of the 3^rd^ century B.C. (Fig. 1b). During the period that immediately preceded the demographic events described in this article (the period between 1^st^ and 2^nd^ centuries B.C.), the lowland of contemporary central Poland was occupied by the Przeworsk culture (Fig. 1c), which replaced the earlier existing PC. The development of the Przeworsk Culture seems to be connected with a Vandal migration. In a similar period, in the regions of Middle Pomerania and Lower Powiśle (zone along the Baltic seashore), the Oksywie culture was established (see Fig. 1c).

The turn of the epochs (i.e., the end of 1^st^ millennium B.C. and the beginning of the 1^st^ millennium A.D.) was connected with further cultural and demographic transformations in the region that is present-day Poland^20^. A new grouping called the Wielbark culture was established, most likely under the influence of Goths and Gepids. To learn more about these processes, we have commenced systematic studies of the populations inhabiting the territory corresponding to present-day Poland in the first centuries AD. Our initial analyses were focused on a large group of individuals living in the region between Oder and Vistula Rivers in the IA (group called the Kow-OVIA)^8,21^. Biological material was collected from a biritual cemetery located in central Wielkopolska (also referred to as Greater Poland - contemporary western Poland) in Kowalewko. Former archeological and anthropological studies showed that approximately 500 people were buried there between 1 and 200 A.D. Our recently published report revealed a high genetic diversity of the Kow-OVIA^8^. Interestingly, women and men from Kowalewko had a significantly different genetic history. In general, the collected data showed how the interactions between newcomers (most likely Goths) and autochthonous communities derived from other cultural traditions could shape the new, local genetic substructure.

This paper is a continuation of our previous work and concentrates on the human population that lived between the Vistula and Bug Rivers (contemporary eastern Poland) in the IA. The studied group is younger than the Kow-OVIA (2^nd^-4^th^ and 1^st^-2^nd^ centuries A.D., respectively) and is believed to represent the next stage of the Goth migrations. The genetic makeup of individuals living at that time in this region has not yet been determined. The biological material was collected from one of the richest IA cemeteries located in east Poland near Masłomęcz village. The cemetery is attributed to the IA Wielbark culture, i.e., to the same culture as the recently characterized site at Kowalewko. This relation in terms of culture and time with geographical separation provided a unique opportunity to obtain the imprint of the demographic processes underlying the genetic variability of the population living during the IA in Central-East Europe. Our analysis involved 27 individuals. We assigned mitochondrial DNA (mtDNA) haplogroups and determined complete mtDNA genome sequences for all of them. We found a high intrapopulation genetic diversity of the studied group. It showed close matrilineal relationship with two other IA populations living, respectively, at the Jutland peninsula and between the Oder and Vistula Rivers. In contrast to our earlier findings concerning Kowalewko (contemporary Western Poland), we found no evidence of distinct genetic history between females and males from Masłomęcz. This observation suggests that both populations were formed or functioned in different ways. Thus, the data presented here provide new insights into the processes that led to the formation of region-specific genetic substructures within the populations inhabiting the Vistula River Basin in the IA.

## Results

### DNA isolation, sequencing and preliminary characteristics of mtDNA

Our studies involved human skeletal remains excavated from the Wielbark culture cemetery of IA. The cemetery is located between the Vistula and Bug Rivers in southeast Poland, close to Masłomęcz village (50°72′39′′N 23°89′27′′E). The studied group, called the Mas-VBIA [abbreviation describing the exact location (Masłomęcz), geographical context (between Vistula and Bug rivers) and time (the IA)], included 27 individuals, whose burial sites previously had been thoroughly characterized archaeologically and anthropologically (Supplementary Table S1). Among others, it was earlier determined that human remains were from 2^nd^ to 4^th^centuries A.D., and the grave furnishings were characteristic of the Goths^22^. Radiocarbon dating of the selected samples (Supplementary Fig. S1 and Table S1) confirmed the archeological estimations.

DNA was isolated from teeth, in a dedicated aDNA laboratory, according to the procedure described by Yang and Svensson^23,24^. In total, 27 DNA samples were obtained. The NGS libraries were successfully generated for all samples. To estimate the amount of human DNA, all of the obtained libraries were subjected to shallow NGS sequencing. In order to determine the amount of endogenous human nuclear DNA and mtDNA content, fastq files were aligned to both GRCh37 and RSRS mtDNA reference sequences. The average coverage of mitochondrial genome was 4.7x and ranged between 0.3x and 22.1 × (Supplementary Table S2). In the case of all samples, human DNA showed typical aDNA damage patterns (C > T and G > A alteration most frequently occurring at the 5′ and 3′ ends of the reads, respectively) (Supplementary Table S3 and Supplementary Fig. 2).

Following the NGS screening, the DNA samples for which average coverage was below 5 for the 95% of the mtDNA genome were subjected to second round of sequencing. Additionally, the DNA samples with endogenous human DNA content <5% were subjected to mtDNA enrichment according to the procedure described by Carpenter^25^ and then sequenced. As a result, we determined the mtDNA haplogroups and full sequences of the mitochondrial genomes for all 27 individuals (Supplementary Table S4). To test for possible human DNA contamination, we used contamMix^26^ to estimate the rates of apparent heterozygosity in mtDNA. The average contamination was approximately 3% (Table 1). All identified haplogroups had been found previously in the populations living in Central Europe from the EBA to the IA, and belonged to 9 haplogroups: H, HV, N1, J, K, T, U, W, and X (Table 1). In addition, based on the anthropological analyses and the raw sequencing data, we determined the sex for 10 and 14 individuals, respectively. In 9 cases sex was assigned with both, anthropological and genetic methods that always gave concordant results (Tables 1 and S4).

**Table 1:** Results of mtDNA haplogroup assignment.

Sample	Haplogroup	Anthropological sex	Genetic sex	mtDNA contamination
PCA0088	U3a1a	M	low chrX and chrY coverage	0.9898451
PCA0089	J1c3	F	F	0.9610449
PCA0090	U3a1a	—	F	0.9935336
PCA0091	U5a1b3	—	M	0.9964154
PCA0092	H16	F	F	0.9970401
PCA0093	T1a9	M	M	0.9048376
PCA0094	HV0f	F	F	0.9726827
PCA0095	H11a1	—	low chrX and chrY coverage	0.9812048
PCA0096	U4c1	—	low chrX and chrY coverage	0.9292417
PCA0097	T2a1a	—	low chrX and chrY coverage	0.988292
PCA0098	H1e2	—	low chrX and chrY coverage	0.9980056
PCA0099	H1cg	—	F	0.9373612
PCA0100	HV0f	M	M	0.9963028
PCA0101	U5a2b3	—	low chrX and chrY coverage	0.9964521
PCA0102	K1c1	M	M	0.9945874
PCA0103	H2a1a	F	F	0.9289728
PCA0104	H1a3	—	low chrX and chrY coverage	0.9814315
PCA0105	U5a2a1	—	F	0.9984908
PCA0106	T2b	—	low chrX and chrY coverage	0.9841855
PCA0107	U5a1b1e	—	low chrX and chrY coverage	0.9879122
PCA0108	T2b23	—	low chrX and chrY coverage	0.9255117
PCA0109	K1a27	—	F	0.9818328
PCA0110	H5e1b	M	M	0.9880837
PCA0111	N1a1a1a2	F	F	0.9538297
PCA0112	T2b2b	—	low chrX and chrY coverage	0.9910141
PCA0113	V	—	low chrX and chrY coverage	0.8358445
PCA0114	H7a1a	—	low chrX and chrY coverage	0.9844894

### Genetic homogeneity of the Mas-VBIA group

**Figure 2:**
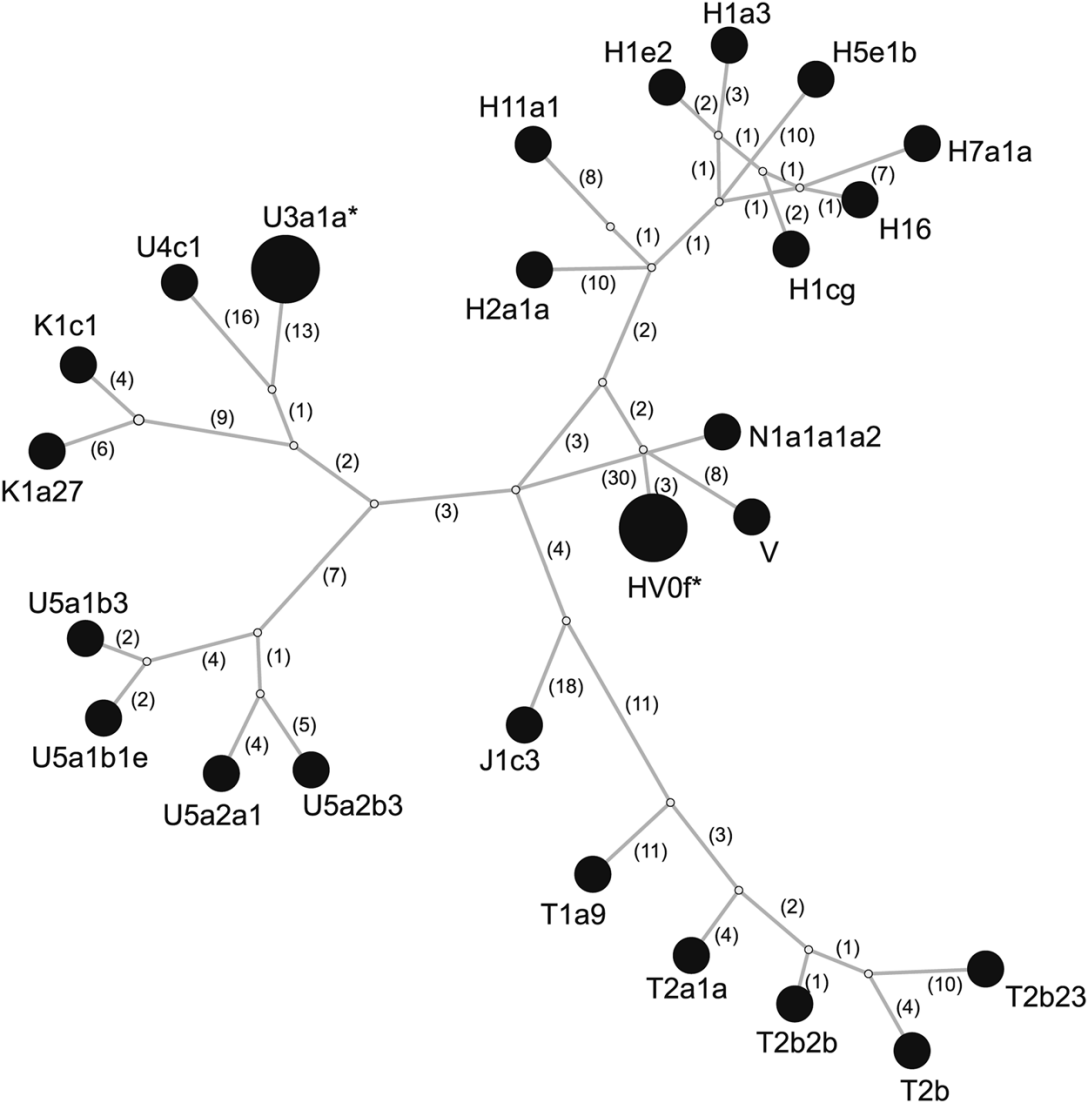
Median Joining Network of 27 individuals from Mas-VBIA based on full mtDNA sequences. Each node corresponds to a haplotype determined for a unique mtDNA sequence. Numbers in brackets show numbers of nucleotide differences between haplotypes. Stars mark haplotypes represented by two individuals with identical mtDNA sequence.

To assess whether the analyzed group of people formed a closed homogenous or opened diversified society, we determined the genetic diversity of the Mas-VBIA. To this end, we compared the full length mtDNA sequences obtained for all 27 individuals (Supplementary Table S4). We found 2 pairs of individuals who had identical mtDNA sequences (PCA0088, PCA0090; PCA0094, PCA0100). Interestingly, persons with the same mitochondrial genomes were buried far from each other (Supplementary Fig. 1). We visualized all mtDNA haplotypes from the MAS-VBIA population with the Median Joining Network (MJN) (Fig. 2).

**Figure 3:**
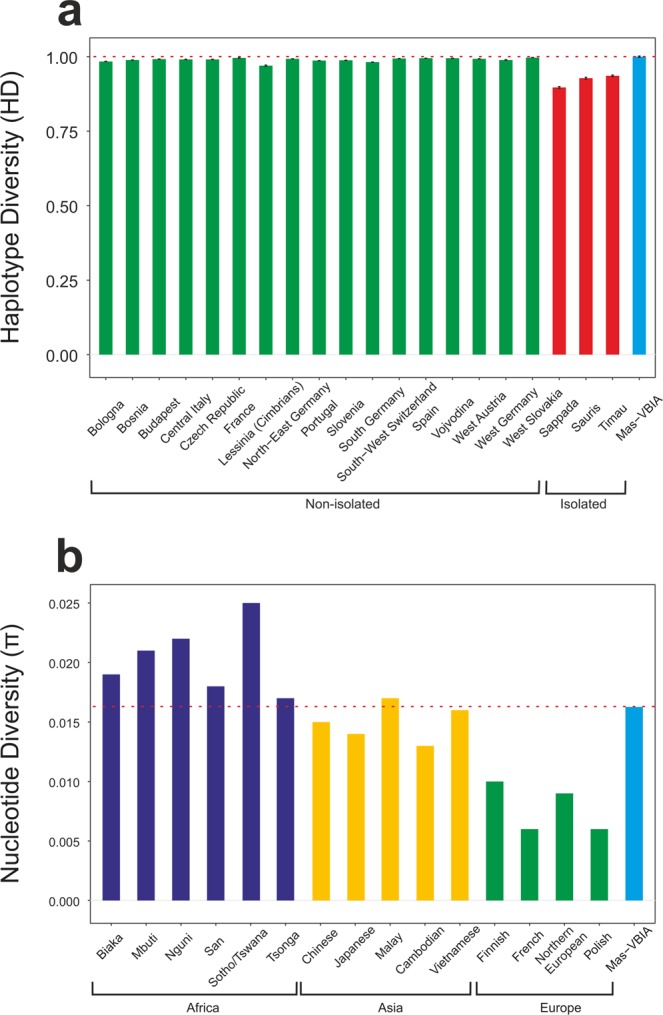
Intrapopulation genetic diversity estimates: (**a)** Haplotype Diversity; (**b)** Nucleotide Diversity. Red dotted line marks the estimate value of diversity of the Mas-VBIA.

Next, using Arlequin ver. 3.5.1 software^27^, we determined the levels of haplotype diversity (HD) based on two fragments of the HVS (hypervariable sequence) region of mitochondrial genome (regions: HVS-I between nucleotide position (np) 16033 and 16365 and HVS-II between np 73 and 340) (Fig. 3a, Supplementary Table S5) and nucleotide diversity (π) based on the fragment of HVS-I region (between np 16000 and 16410) (Fig. 3b, Supplementary Table S6). The HD level calculated for the Mas-VBIA (1.0000 +/− 0.0113) was in the range of high values currently observed for the contemporary, non-isolated European populations. The level of π determined for the Mas-VBIA (0.016263 +/− 0.008846) was high and outside the range of values reported for the present-day European populations (0.009) and in the range of the values reported for contemporary Asian populations^28^. Thus, the results of all three analyses of MJN, HD and π showed that the Mas-VBIA was a genetically highly diverse population.

To exclude the possibility that the presence of two potentially maternally related individuals in the relatively small tested group will affect the results of our consecutive analyzes, we removed the two individuals from the tested group - one from each pair with identical mtDNA sequences.

### Matrilineal ancestry of the Mas-VBIA and its contribution to the demographic history of Europe

#### mtDNA haplogroup frequency

**Figure 4:**
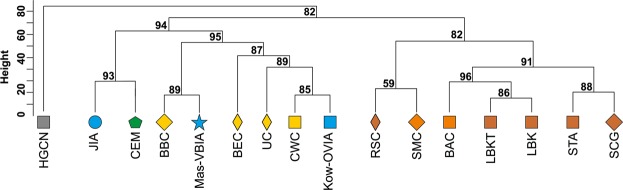
Unsupervised hierarchical clustering with Ward method and Manhattan distance of haplogroup frequencies for the CEPT populations. P-values of the clusters are given as the percent of reproduced clusters based on 10,000 bootstrap replicates. Symbols indicate populations from Central Europe (squares and diamonds), Southern Scandinavia and Jutland Peninsula (circles), and East Europe/Asia (stars). Color shading of data points denotes to Hunter-Gatherers (grey), Early Neolithic (brown), Middle Neolithic (orange), Late Neolithic/Early Bronze Age (yellow), Iron Age (blue), and the present-day Central Europe Metapopulation (CEM, green). Abbreviations: Central/North European Hunter-Gatherers (HGCN), Starčevo Culture population (STA), Linearbandkeramik in Transdanubia (LBKT), Linearbandkeramik population from Central Europe (LBK), Rössen Culture (RSC), Schöningen Group (SCG), Baalberge Culture (BAC), Salzmünde Culture (SMC), Bernburg Culture (BEC), Corded Ware Culture (CWC), Bell Beaker Culture (BBC), Unetice Culture (UC), Jutland Iron Age (JIA), Kowalewko Oder and Vistula Iron Age (Kow-OVIA), Masłomęcz Vistula and Bug Iron Age (Mas-VBIA).

mtDNA haplogroup H was the most common in the Mas-VBIA (32%); T2 and U5a were the second most common (16% each). To place the Mas-VBIA in the Central European space-time, we compared mtDNA haplogroup frequencies within the set of populations that lived in this region from the Mesolithic until the present (Central European Population Transect (CEPT), for details, see Materials and methods, Tables 2 and S7–S8). The results of unsupervised hierarchical clustering (Ward’s method with Manhattan distance) of the CEPT (Fig. 4) were generally compatible with the known chronology of the formation of the genetic structure of the Central European population. The first to be separated from other populations were the Hunter Gatherers from Central and North Europe (HGCN). Next, we observed a divergence of the studied populations into 2 groups. The first one consisted of Early Neolithic (EN) populations [Starčevo culture (STA), Linearbandkeramik in Transdanubia (LBKT), Linearbandkeramik population from Central Europe (LBK), Rössen culture (RSC), Schöningen Group (SCG)] and MN populations [Baalberge culture (BAC), Salzmünde culture (SMC)]. The second group consisted of LN/EBA populations [BEC (Bernburg culture), CWC, Bell Beaker culture (BBC), Unetice culture (UC)] and post-EBA populations [Jutland peninsula population from IA (JIA), Kow-OVIA, Mas-VBIA and Central European Metapopulation (CEM)]. Within this group, the JIA and CEM formed a separate sub-clade (alpha = 0.93). In addition, the remaining LN/EBA populations split into the two sub-clades. The first one was formed by the Mas-VBIA and BBC and the second by the BEC, UC, CWC and Kow-OVIA.

**Table 2:** Published reference ancient mtDNA data and populations abbreviations.

Abbreviation	Population	Time period
HGCN	Hunter Gatherers Central North Europe	15400–4300 BP
HGSW	Hunter Gatherers South West Europe	12000–5000 BP
HGE	Hunter Gatherers Eastern Europe	15000–4500 BP
STA	Starčevo culture	8200–7450 BP
LBKT	Linearbandkeramik culture in Transdanubia	7600–6900 BP
LBK	Linearbandkeramik culture in Central Europe	7500–6900 BP
RSC	Rössen culture	6600–6200 BP
SCG	Schöningen group	6100–5900 BP
BAC	Baalberge culture	5900–5400 BP
SMC	Salzmünde culture	5400–5100 BP
BEC	Bernburg culture	5100–4600 BP
CWC	Corded Ware culture	4800–4200 BP
BBC	Bell Beaker culture in Central Europe	4500–4200 BP
UC	Unetice culture	4200–3150 BP
PWC	Pitted Ware Culture	5200–4300 BP
CAR	Cardial/Epicardial culture of the Iberian Penisula	7400–5700 BP
NPO	Portuguese Neolithic population	7200–5000 BP
NBQ	Neolithic population from Basque Country and Navarre	8100–7100 BP
TRB	Funnel Beaker culture	6300–4800 BP
TRE	Treilles culture	7000 BP
BAS	Bronze Age Siberia	4400–2800 BP
BAK	Bronze Age Kazakhstan	4000–2600 BP
RRBP	Gurgy ‘Les Noisats’ group	7000–6000 BP
MIR	Iberian Chalcolithic El Mirador Burgos individuals	4500–4050 BP
JIA	Jutland Iron Age	2500 BP - 400 AD
IIA	Iberian Iron Age population	2800 BP - 50 AD
SCY	Iron Age Scythian samples	2300–2600 BP
SSP	Scytho-Siberian Pazyryk Culture	2400–2300 BP
YAM	Yamnaya culture	5300–4600 BP
Kow-OVIA	Oder Vistula Iron Age	0–200 AD
Mas-VBIA	Vistula Bug Iron Age	100–300 AD

**Figure 5:**
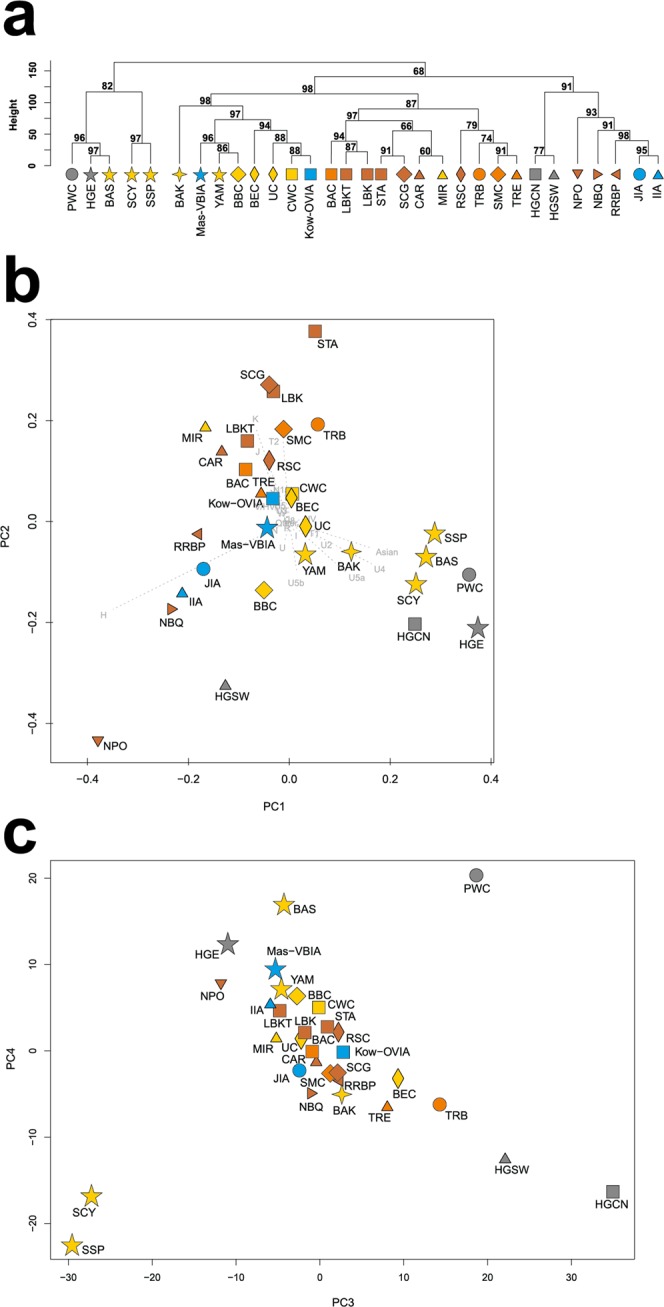
(**a**) Unsupervised hierarchical clustering with Ward method and Manhattan distance of haplogroup frequencies for the EPT populations. P-values of the clusters are given as the percent of reproduced clusters based on 10,000 bootstrap replicates; (**b**) Principal components 1 and 2 of the PCA on the haplogroup frequencies of EPT populations. Symbols indicate populations from Central Europe (squares and diamonds), Southern Scandinavia and Jutland Peninsula (circles), Iberian Peninsula (triangles), and East Europe/Asia (stars). Color shading of data points denotes to Hunter-Gatherers (grey), Early Neolithic (brown), Middle Neolithic (orange), Late Neolithic/Early Bronze Age (yellow) and Iron Age (blue). The first two principal components of the PCA display 48.4% of the total genetic variation. Each haplogroup was superimposed as component loading vectors (grey dotted lines) proportionally to their contribution. Abbreviations: Central/North European Hunter-Gatherers (HGCN), Southwestern European Hunter-Gatherers (HGSW), East European Hunter-Gatherers (HGE), Starčevo Culture population (STA), Linearbandkeramik in Transdanubia (LBKT), Linearbandkeramik population from Central Europe (LBK), Rössen Culture (RSC), Schöningen Group (SCG), Baalberge Culture (BAC), Salzmünde Culture (SMC), Bernburg Culture (BEC), Corded Ware Culture (CWC), Bell Beaker Culture (BBC), Unetice Culture (UC), Pitted Ware culture (PWC), Funnel Beaker culture (TRB), Jutland Iron Age (JIA), Cardial/Epicardial culture of the Iberian Peninsula (CAR), Portuguese Neolithic population (NPO), Neolithic population from Basque Country and Navarre (NBQ), Iberian Chalcolithic El Mirador Cave individuals (MIR), individuals from Iberian Iron Age period (IIA), Treilles Culture (TRE), Gurgy ‘Les Noisats’ group (RRBP), Bronze Age Kurgan samples from South Siberia (BAS), Bronze Age Kazakhstan (BAK), Yamnaya (YAM), Iron Age Scythian (SCY), Scytho-Siberian Pazyryk Culture (SSP), Kowalewko Oder and Vistula Iron Age (Kow-OVIA), Masłomęcz Vistula Bug Iron Age (Mas-VBIA); (**c**) Principal components 3 and 4 of the PCA on the haplogroup frequencies of EPT populations.

To look wider on the Mas-VBIA ancestry and its relationship with other contemporary populations, we assembled selected ancient populations in the European Population Transect dataset (EPT) (for details, see Materials and methods, Tables 2 and S7), which, as previously described, was subjected to hierarchical clustering and additionally to PCA of the mtDNA haplogroup frequencies (Fig. 5a–c, Supplementary Table S9). The results of EPT clustering were consistent with those obtained for the CEPT. The Mas-VBIA was placed within central European LN/EBA group and was related closely with the BBC and YAM. The Kow-OVIA was also placed within the central European LN/EBA group but formed a clade with the CWC, UC and BEC (Fig. 5a). First, the two principal components of the PCA plot (Fig. 5b) positioned the Mas-VBIA near the center of the graph, closest to the Kow-OVIA but also close to the CWC, BEC, UC and YAM. The 3rd and 4th principal components (Fig. 5c) placed the Mas-VBIA in close proximity to the YAM and BBC and revealed the Kow-OVIA’s higher affinity to the BEC, CWC and UC, as supported by the hierarchical clustering.

To visualize the relationships of the Mas-VBIA group with contemporary humans, we placed it on a map showing the matrilineal genetic structure of present-day populations. The PCA of haplogroup frequencies observed in the Mas-VBIA and 73 extant worldwide populations (Supplementary Fig. S3, Supplementary Table S10) showed that the Mas-VBIA was located within a range of European genetic diversity.

#### Genetic distances

The relationships between the Mas-VBIA and the earlier and later European populations were also determined by measuring the genetic distances among them. Genetic distances were determined based on a fragment of the mtDNA HVS-I sequence (the region between np 16064 and 16400) (Supplementary Table S11). In the first step, we concentrated on the CEPT (populations from Central Europe from Mesolithic to present-day). The established fixation indexes (Fst) showed that the Mas-VBIA was most closely related to the BEC (Fst = 0, p = 0.7608) and RSC (Fst = 0, p = 0.6529). Both the BEC and RSC share a common archaeological history connected with the spread of the TRB culture. Similarly to the Mas-VBIA, the Kow-OVIA had small genetic distances to the BEC (Fst = 0, p = 0.748) and RSC (Fst = 0.0002, p = 0.567). Unlike the Kow-OVIA, which was closely linked with the JIA, the Mas-VBIA had smaller genetic distances to the LN CWC and BBC populations (Fst = 0, p = 0.5872; Fst = 0, p = 0.67342, respectively) than to the JIA (Fst = 0.00915, p = 0.3768).

**Figure 6:**
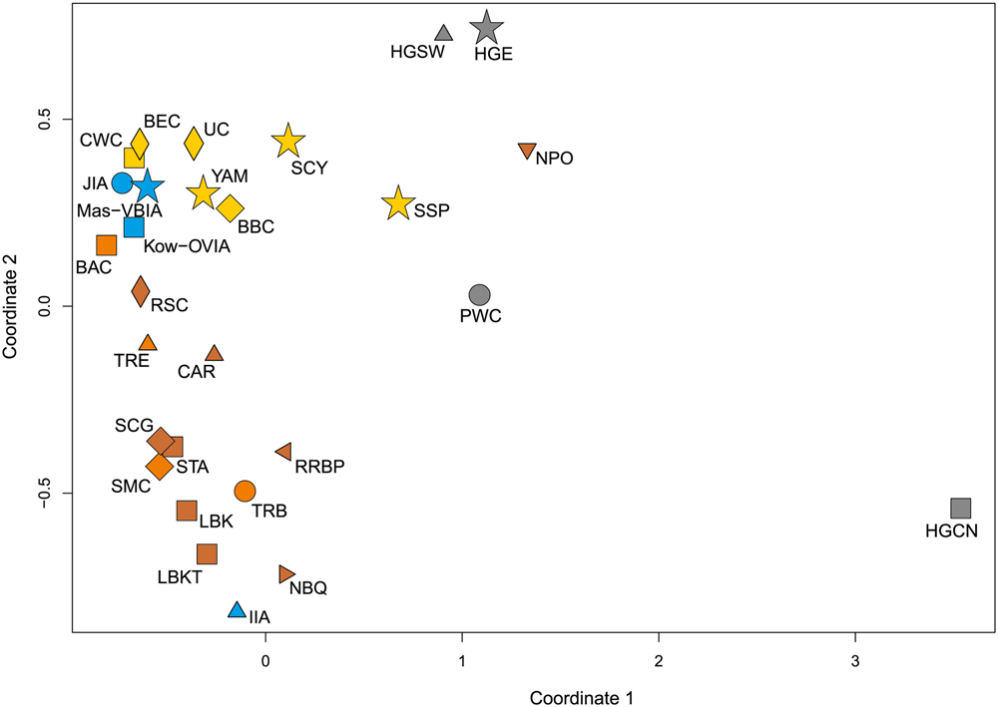
MDS plot of Slatkin’s Fst values for EPT populations. Fst values were obtained for mtDNA HVS-I region (np 16064–16400).

In the second step, we focused on the populations living in all of Europe from the Mesolithic to the IA. To this end, we selected 27 populations from the EPT, for which sequences of the mtDNA HVS-I region between np 16064 and 16400 (Table S12) were known for a considerable number of individuals. The calculated genetic distances (Supplementary Table S12) showed once again that the Mas-VBIA was the closest to the BEC (Fst = 0, p = 0.7418). Furthermore, the close connection to the Funnel Beaker cultural horizon was recapitulated with the low genetic distance between the Mas-VBIA and the TRB (Fst = 0, p = 0.6028). A small genetic distance was also found between the Mas-VBIA and the YAM (Fst = 0, p = 0.87892). At the same time, the genetic distance between the Mas-VBIA and the LBK was high (Fst = 0.02949, p = 0.0634). The results of the genetic distance analysis were visualized on the multidimensional scaling (MDS) plot (Fig. 6). In accordance with the established genetic distances, the MDS located the Mas-VBIA, CWC, BBC, BEC and YAM close together.

### The Mas-VBIA in the context of the processes shaping the genetic structure of the Central European population

In the next stage of our studies, we attempted to determine how the Mas-VBIA fits into the existing scheme describing the process of the formation of the genetic structure of the Central European population. For this purpose, we performed an Analysis of Molecular Variance (AMOVA). In the first step, we determined an optimal division of the populations from CEPT, excluding the HGCN and CEM. The division was considered to be optimal if the intragroup variability (Fsc) was minimal and intergroup variability (Fct) was maximal. Considering earlier observations made by other authors^29,30^, we allocated all EN/MN populations (STA, LBKT, LBK, RSC, SCG, BAC, SMC) to one group because of their high genetic homogeneity and analyzed 41 different combinations of populations’ groupings (Supplementary Table S13). We found that Fsc and Fct were optimal if the studied populations were divided into three groups: (STA, LBKT, LBK, RSC, SCG, BAC, SMC) + (CWC, UC, JIA) + (BEC, BBC, Mas-VBIA, Kow-OVIA). Importantly, we observed that the groupings were always better when the Mas-VBIA and Kow-OVIA were in the same group. This observation was not biased by the batch effect as samples from both cemeteries were sequenced separately. Moreover, particular samples were sequenced in multiple runs and in two independent laboratories. The obtained results suggest that the JIA had close relationships with the North-Central Europe populations (CWC and UC), whereas the Mas-VBIA was linked to the Funnel Beakers (e.g., BEC) and Bell Beakers.

Next, to determine to what extent particular populations contributed to the genetic structure of the contemporary Central European population, we performed an analogous analysis, but the studied group was extended by the CEM (CEPT with excluded HGCN). Once again, the populations of the EN/MN (STA, LBKT, LBK, RSC, SCG, BAC, SMC) were treated as one group, and we analyzed 91 combinations of the other fossil populations and the CEM (Supplementary Table S14). The combination of 3 groups (STA, LBKT, LBK, RSC, SCG, BAC, SMC) + (BEC, UC) + (CWC, BBC, Mas-VBIA, Kow-OVIA, JIA, CEM) showed the best optimization of Fsc and Fct values. Furthermore, clustering was always better if the CEM and IA populations did not form a separate group but were placed together with the CWC or BBC.

The results of the above analyses were consistent with our previous observations^8^ suggesting that the contribution of the particular populations to the genetic structure of present-day Europe is not fully consistent with the chronology according to which these populations inhabited Central Europe. For example, the Mas-VBIA, Kow-OVIA, JIA, BBC and CWC contributed more significantly to the genetic structure of present-day Europe than the UC.

### mtDNA lineages

To get better insight into the processes that shaped the genetic structure of the studied population, we determined which populations gave rise to the haplotypes found in the Mas-VBIA (Supplementary Table S15). Our analysis showed that 44% of the mtDNA haplotypes came from EN populations. In this group, mtDNA haplogroups H and T2 were most frequent. Haplotypes that appeared in the LN and EBA were not frequently detected in the Mas-VBIA (12% and 6%, respectively).

A consecutive analysis of shared haplotypes^31^ (Supplementary Table S16) revealed that 78% of the haplotypes inherited by the Mas-VBIA from the LN/EBA or earlier populations were present in at least 1 LN/EBA population (BEC, CWC, BBC or UC), and 28% were present in all three CWC, BBC and UC populations. It was noteworthy that neither of the haplotypes inherited by the Mas-VBIA was uniquely shared only with the BEC. All haplotypes shared by the BEC and Mas-VBIA were also present in each of the remaining LN/EBA populations. Interestingly, though the Mas-VBIA showed a high genetic distance to the UC, it shared more haplotypes with the UC than with any other LN/EBA population. Additionally, the haplotypes shared uniquely with only one LN/EBA population were most frequently observed for the Mas-VBIA and UC. Here, however, it should be noticed that the UC is represented by a high number of individuals; thus, the chance of finding shared haplotypes was higher. Twenty-one percent of the haplotypes found in the Mas-VBIA were absent in any LN/EBA population.

## Discussion

Despite intensive progress in genomic studies of ancient European populations, our knowledge on demographic processes that occurred in the Central-Eastern part of the continent after the Neolithic is still very limited. Until recently, there were no data describing the genetic makeup of people living in Central Europe east of the Oder River during the BA and IA. Archaeological and genomic findings suggested that on the turn of the LN and BA this region (contemporary Poland) was influenced by four major cultures: BBC, CWC, UC and YAM (Fig. 1a). However, due to the lack of reliable data, the actual range of these cultures, and consequently, the placement of the borders between them, as well as the later history of these populations, are difficult to determine.

To extend our knowledge on the issues described above, we initiated broad genetic studies of the populations inhabiting the territory of present-day Poland in the IA. In our earlier paper, we characterized the maternal genetic makeup of the individuals living in the west part of contemporary Poland^8^. In this report, we focused on the maternal genetic history of the Mas-VBIA group that lived in the eastern part of contemporary Poland (the region located between the Vistula and Bug Rivers) during the 2^nd^ to 4^th^ century A.D. We found that the intrapopulation diversity of the Mas-VBIA (π = 0.016263) was beyond expectations. It was as high, as is currently observed for Asian populations (π between 0.013 and 0.017), and exceeded the values typical for non-isolated European populations (π between 0.006 and 0.01), as well as the intrapopulation diversity previously determined for its western counterpart – the Kow-OVIA (π = 0.0079). However, it should be noted here that due to the postmortem DNA damage, the diversity estimates could be inflated. Accordingly, one can hypothesize that people living between the Vistula and Bug Rivers in the IA formed genetically diversified, non-isolated populations. The haplogroup frequencies observed for the Mas-VBIA were similar to those reported for the Kow-OVIA. Haplogroup H frequency was intermediate between CWC and BBC and lower than in CEM. Interestingly, the frequencies of the mtDNA haplogroups U5a (characteristic for Eastern Hunter Gatherer groups) and U5b (characteristic for Western Hunter Gatherer groups) were a significantly different in the Mas-VBIA and other IA populations studied so far (the JIA and Kow-OVIA). The Mas-VBIA was characterized by the absence of mtDNA haplogroup U5b and the high frequency of mtDNA haplogroup U5a (14.8%), whereas in the JIA and Kow-OVIA, mtDNA haplogroup U5b was more prevalent than U5a. As noticed previously^8^, the domination of U5b over U5a in Central Europe was observed only during the IA. Therefore, either the process that led to the temporal increase of U5b mtDNA haplogroup frequency did not affect regions east of Vistula, or its effects were subsequently removed by another demographic event.

All high dimensional analyses of genetic diversity (PCA, hierarchical clustering, MDS) within the Mas-VBIA and other ancient and extant populations were consistent and showed that the Mas-VBIA was closely connected with the Kow-OVIA and had a similar genetic structure to that of Central European LN/EBA populations. Within the group of three IA Central European populations, the Mas-VBIA was the closest related with the Kow-OVIA, and the distance between the JIA and Mas-VBIA was shorter than that between the JIA and Kow-OVIA. Furthermore, the Mas-VBIA had close genetic connections with the YAM, located in the eastern parts of Europe. We did not observe these connections in either the Kow-OVIA or the JIA. Earlier, Haak *et al*.^32^ showed that there are clear genetic links, even at the mtDNA level, between the central European CWC and BBC populations and the YAM. However, if close genetic connections between the Mas-VBIA and YAM were a result of the LN migratory events, the same should be observed in the remaining IA populations of the region. Therefore, we propose that the presence of the Pontic-Caspian steppe genetic component in the Mas-VBIA is a result of the migratory event or events that occurred in the IA period. Considering the high prevalence of the mtDNA haplogroup U5a in the Mas-VBIA, one can hypothesize that the direction of this movement was from east to west.

Another interesting observation was the genetic proximity between the BEC and virtually all subsequent populations (the CWC, BBC, UC, JIA, Kow-OVIA and Mas-VBIA). This proximity was most evident when shared mtDNA haplotypes were analyzed. We found that the haplotypes shared between all LN and IA populations were nearly always present in the BEC. The BEC is part of a TRB cultural horizon that spread from North to Central Europe during the MN in the form of the sequence of Danubian populations^14^. Thus, here we provide an additional line of evidence that Danubian Neolithic populations were a common genetic background of all Central-North and Central-East populations in Europe.

**Figure 7:**
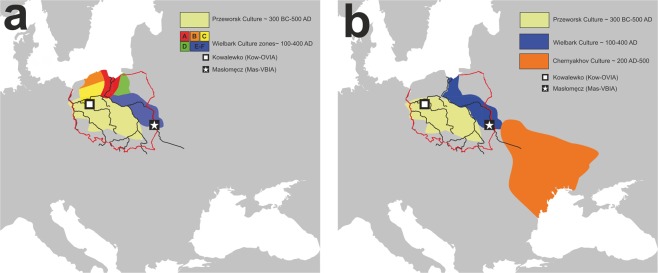
Geographical distribution of the archaeological cultures in contemporary Poland linked to the early migrations of the Goths: (**a)** formation stages of the Wielbark culture; (**b)** late stage of the Wielbark culture. Europe map by Roke, retrieved from https://commons.wikimedia.org/wiki/Category:Blank_maps_of_Europe#/media/File:BlankMap-Europe-v3.png, used under Creative Commons Attribution-ShareAlike 3.0 Unported (https://creativecommons.org/licenses/by-sa/3.0/deed.en), modified with Corel Draw ver. 12.0.

There are two major historical narratives describing the formation of the Wielbark culture. The first one links its development with Goth migrations^33^, and the second, with the spread of the local Oksywie culture that appeared at the Baltic seashore at approximately the 2^nd^ century B.C^20,34^. In addition, there are at least two hypotheses concerning the origin of Goths. One of them assumes that the homeland of the Goths was located in the southernmost part of the Germanic territories other than in Scandinavia^35^. Considering the results obtained for both the Kow-OVIA and Mas-VBIA, one can assume that they are, to a large extent, consistent with the postulated chronology of early migrations of Goths and their settlement in Central-East Europe. The high genetic diversity of the Mas-VBIA strongly corresponds with the suggested role of Masłomęcz at that time^22^. The archaeological findings indicate that in the 2^nd^ and 3^rd^ centuries A.D., it was one of the major cultural and political Goth centers. In addition, the genetic relationships reported here between the Mas-VBIA and both earlier characterized IA populations (the Kow-OVIA and JIA) support the opinion that southern Scandinavia was the homeland of the Goths. According to some authors, the process of their migration through the contemporary territory of Poland, which was connected with the spread of the Wielbark culture, can be divided into the following 6 stages, named with the letters A-F (for details, see Fig. 7a)^20^. In the first stage, the Goths colonized the mouth of the Vistula River during the 2^nd^ and 1^st^ centuries B.C. (stage A). Then, they spread west along the Baltic seashore (stage B). In stage C, the Goths moved south and ousted the Przeworsk culture from the northwest part of contemporary Poland, including Kowalewko (1^st^ and 2^nd^ centuries A.D.). The Goth’s settlements established in this area could have a rather temporal character suggested by the fact that the genetic structure of the Kow-OVIA population was biased by sex. Males from the Kow-OVIA were closely related with the JIA and females with Neolithic farmers^8^ (most likely they represented the local population related with the Przeworsk culture). During the next stage (D), the Goths spread east of the Vistula, and then they migrated along the Vistula and Bug Rivers towards the Black See and established the Chernyakhov culture by mixing with Pontic–Caspian steppe populations (stage E). Finally, a part of the Chernyakhov culture population moved back and established a large settlement near Masłomęcz called the “Masłomęcz group” (stage F, 2^nd^ and 3^rd^ centuries A.D.) (Fig. 7b). Such a scenario explains a large part of our findings well, including (i) the high prevalence of the mtDNA U5a haplogroup (characteristic for the eastern parts of Europe); (ii) the close genetic distance to the YAM observed for the Mas-VBIA and the lack of these characteristics in the cases of the Kow-OVIA and JIA; and (iii) the high genetic diversity of the Mas-VBIA. The latter indicates that the “Masłomęcz group”, often considered the Goths, from a genetic standpoint, was a mixture of different populations. Unfortunately, the presented data cannot unequivocally verify both hypotheses on the Goth origin and their relationships with the Oksywie culture. Thus, we still do not know whether the Goths replaced the Oksywie culture or induced its formation. It is also possible that all the scenarios presented here contain some truth and describe different periods of the Goth population development and migrations. Thus, further studies are necessary to verify them.

## Materials and Methods

### aDNA extraction and library preparation

For the purposes of this study, 27 samples (teeth) were obtained from 27 individuals from the Masłomęcz archeological site (Supplementary Table S1). After being transported to a dedicated aDNA laboratory (at the Institute of Anthropology, Faculty of Biology, Adam Mickiewicz University, Poznan, Poland), the teeth were cleaned with 5% NaOCl and rinsed with sterile water, followed by UV irradiation (254 nm) for 2 hours for each site. The roots of the teeth were drilled using Dremel® drill bits. Bone powder (~250 mg) was digested with proteinase K, and DNA-containing extract was purified with the use of the silica-based method following Yang and Malmstrom^23,36^. Genomic libraries were prepared following the protocol of Meyer^37^, with omission of the initial sonication step due to natural fragmentation of aDNA. In order to maximize the chance of amplification of each unique DNA template molecule, six separate PCR reactions were set up for each library. PCR amplifications were performed in 25 µl, with 3 µl of the DNA library template, 12.5 µl of 1x AmpliTaq Gold® 360 Master Mix (Life Technologies), 0.5 µl of indexing primer (10 µM) and 0.5 µl of PCR primer IS4 (10 µM)^38^. The PCR profile was as follows: initial denaturation (94 °C, 12 min), 12–16 cycles of 94 °C (30 s), 60 °C (30 s), 72 °C (45 s) and final extension (72 °C, 10 min). PCR reactions for the same library were pooled and purified with AMPure® XP beads (Agencourt-Beckman Coulter)^38^. The quality and size distribution of the libraries were verified with a High Sensitivity DNA kit and 2100 Bioanalyzer system (Agilent), while the DNA concentration was determined with a Qubit fluorimeter and Qubit dsDNA HS Assay Kit (ThermoFisher Scientific), according to the manufacturer’s protocols.

### Next generation sequencing (NGS) and enrichment of aDNA libraries

To screen the libraries, shallow NGS sequencing with the Genome Analyzer GAIIx (Illumina, California, USA) and TruSeq SBS Kits v5-GA (Illumina) was applied. Seven to eight libraries were pooled per lane. On average, 6.3 mln 75 bp-long reads were collected per library. Libraries with small human DNA content below 5% were enriched in mtDNA or whole human genome sequences using MYbaits (Arbor Biosciences, Michigan, USA) and an in-solution hybridization capture procedure, according to the recommendations of the manufacturer. mtDNA enrichment was performed with MYTObaits (Arbor Biosciences), a set of 80-bp long tiling probes designed based on rCRS (Cambridge Reference Sequence). Two rounds of enrichment were applied, each followed by 14 amplification cycles. Enriched libraries were sequenced using GAIIx (Illumina) again. On average, 6 mln 75 bp-long reads were collected per mtDNA-enriched library and human genome content was in the range of 55–74% (Supplementary Table S2).

### Filtering, mapping and variant calling

Raw sequencing data (fastq files) were filtered with AdapterRemoval^39^ by trimming missing nucleotides from both ends with the threshold of minimum quality of 30 and minimum length of 25 nucleotides. Filtered reads were aligned with BWA ver. 0.7.10^40^ to the rCRS mitochondrial and GRCH 37 reference genomes, with the seed blocked for higher sensitivity and other parameters set as default, as suggested by Schubert^41^, with the command *bwa aln -l 1000 reference*.*fasta input*.*fastq*. To cope with the effects of DNA damage, we trimmed 3 bases from both read ends. Following the alignment, duplicate reads were removed with picard-tools ver. 1.117 MarkDuplicates. Read depth and coverage were assessed with samtools ver. 1.2^42^ and bedtools ver. 1.2^43^, respectively. Consensus fasta sequences for haplogroup prediction and sequence analyses were generated with FreeBayes ver. 1.0.2–33-gdbb6160. To assemble complete consensus mtDNA sequences we applied the following quality requirements: minimum coverage per base >=3, missing nucleotide count < 5%, no missing nucleotides in the HVS-I sequence, base call supported by the 3/5 majority of reads^44^ (Supplementary Table S4).

### Human DNA damage patterns

To examine data authenticity, we used mapDamage 2.0^45^ and estimated whether the human DNA damage parameters for each sample were typical for aDNA: (i) λ, the fraction of nucleotides positioned in single-stranded DNA overhangs context, (ii) δs, C → T deamination probability in the single-stranded overhangs context, and (iii) δd, C → T deamination probability in the double-stranded DNA context (Supplementary Table S3).

### Contamination assessment

To estimate the level of contamination with contemporary human DNA or with other human aDNA, we used the software contamMix^26^. According to its authors, the software uses 311 present-day human mtDNA sequences as potentially contaminating population and estimates the contamination by comparing the alignment rates between a particular sample’s consensus mtDNA sequence and whole mtDNA sequences of 311 potential contaminants.

### Genetic sex estimation

Genetic sex of each individual was estimated using the Ry method as described by^46^. The method is based on dividing the number of sequences mapped to the Y chromosome to the number of those mapped to X and Y chromosomes. Only sequences with mapping quality of minimum 30 were considered. Sex assignment was performed for samples with at least 3000 reads aligned to the sex chromosomes.

### Analysis of intrapopulation genetic diversity

Haplotype diversity (HD) was analyzed with Arlequin ver. 3.5.1^27^ on two fragments of mtDNA HVS (HVS-I, located between nucleotide positions (np) 16033–16365, and HVS-II np 73–340) (Supplementary Table S5). Nucleotide diversity (π) was calculated for the fragment of mtDNA HVS-I (np 16000–16410) (Supplementary Table S6). Furthermore, we visualized the intrapopulation diversity of the Mas-VBIA using the Median Joining Network (MJN) method on 27 full mtDNA sequences (Fig. 2).

### mtDNA haplogroup frequency analyses

Haplogroups were assigned based on complete mtDNA sequences using HaploFind^47^ with respect to Phylotree build 17 (http://www.phylotree.org/)^48^. Only samples with a haplogroup score ≥0.8 and average coverage ≥3 were used in downstream analyses. To assess changes in temporal mtDNA haplogroup frequency from the Mesolithic to the present day, we performed Ward clustering with Euclidean distance on 23 haplogroups (H, H5, HV, HV0, V, I, J, K, N, N1a, R, T1, T2, U, U2, U3, U4, U5a, U5b, U8, W, X and other). We used a set of 27 samples from this study and 14 ancient populations from Central/North Europe (Supplementary Table S7), and a generated Central European Metapopulation (CEM) composed of 500 random individuals sampled from extant populations of Poland, the Czech Republic, Germany and Austria, as in^30^. We called this group CEPT (Supplementary Table S8).

To compare the mtDNA variability of the Mas-VBIA in a broader geographical context, we also applied unsupervised hierarchical clustering with the Ward method and Euclidean distance and a Principal Components Analysis (PCA) on haplogroup frequencies of the Mas-VBIA and published ancient mtDNA data of populations from across Europe and West Asia. We called this group EPT (European Population Transect) (Supplementary Table S7). Haplogroups were divided into 24 groups present in ancient individuals (Asian [A, C, D, F, G, Z], N, N1a, I, J, W, X, R, HV, V/HV0, H, H5, T, T1, T2, J, U, U2, U3, U4, U5a, U5b, U8, K and other) (Supplementary Table S9).

To elucidate affinities of our samples in relation to present day populations, we performed a PCA analysis based on 23 haplogroup frequencies (Asia [A, C, D, F, G, Z], Africa [L], N1a, I, I1, W, X, HV, V/HV0, H, H5, T1, T2, J, U, U2, U3, U4, U5a, U5b, U8, K and other), with public data from 73 extant populations (Supplementary Table S10).

Cluster significance was tested by performing 10,000 permutations with the pvclust package in R ver. 3.3.0. A PCA was conducted with prcomp of the vegan package in R ver. 3.3.0 (http://R-project.org).

### Genetic distance analyses

For sequence-based analyses, the longest mtDNA HVS-I fragment present in the biggest fraction of published samples was selected (np 16064–16400). Additionally, for newly reported samples from this study, no missing nucleotides were allowed in the selected HVS-I range, and at least 3x coverage was expected for 95% of the nucleotides. To examine genetic affinities on the sequence level, we calculated genetic distances (Fst)^49^ between two sample sets: CEPT (Supplementary Tables S11 and S17) and EPT (Supplementary Tables S12 and S18), including only those individuals for which the 16064–16400 fragment of mtDNA HVS-I was present.

Pairwise and Slatkin’s Fst^50^ values were calculated in Arlequin ver. 3.5.1 for both datasets separately, with the associated substitution model and gamma values selected with jModel test 0.1 AIC and BIC^51^. P-values were calculated by performing 10,000 permutations. Genetic distances were visualized on an MDS plot with the metaMDS function from the vegan package in R ver. 3.3.0.

### Analysis of genetic structure

To examine whether genetic affinities between particular populations from CEPT were a result of a shared genetic structure, we have conducted an Analysis of Molecular Variance (AMOVA) in 41 combinations of CEPT populations (excluding HGCN and CEM) (Supplementary Table S13). Next, we tested the genetic contributions of ancient populations to the extant mtDNA variability by analyzing AMOVA results from 91 combinations of CEPT populations (excluding HGCN) (Supplementary Table S14). Statistical significance was obtained by performing 10,000 permutation tests. An associated substitution model and gamma values were calculated separately for both scenarios with jModel test 0.1 AIC and BIC^51^ (see Supplementary Information).

### mtDNA lineage analyses

To further test genetic affinities and account for the temporal succession of archaeological cultures in Central Europe, we conducted an analysis of shared ancestral haplotypes as described in^29^ in CEPT (Supplementary Table S15), and a classical shared haplotype analysis^31^ in LN/EBA and IA populations (Supplementary Table S16).

## Supplementary information

Supplementary Tables

Supplementary information

## Data Availability

The 27 novel complete mtDNA sequences supporting the results of this article are available in the National Center for Biotechnology Information (Genbank) under accession numbers MH492638-MH492664, http://www.ncbi.nlm.nih.gov/Genbank/.
