# Supplementary material for: Goth migration induced changes in the matrilineal genetic structure of the central-east European population
Source: Sci Rep. 2019 May 1;9:6737. doi: 10.1038/s41598-019-43183-w (PMC6494872; doi:10.1038/s41598-019-43183-w)
Supplement: Supplementary file 2 — Supplementary information [file 41598_2019_43183_MOESM2_ESM.pdf]

Supplementary Materials for:

Goth migration induced changes in the matrilineal genetic structure of the central-east European population

I. Stolarek[1], L. Handschuh[1], A. Juras[2], W. Nowaczewska[3], H. Kocka-Krenz[4], A. Michalowski[4], J. Piontek[2], P. Kozlowski[1], M. Figlerowicz[1,5]*

[1] Institute of Bioorganic Chemistry, Polish Academy of Sciences, Poznan, Poland.

[2] Institute of Anthropology, Faculty of Biology, Adam Mickiewicz University, Poznan, Poland.

[3] Department of Human Biology, Faculty of Biological Sciences, Wroclaw University, Wroclaw, Poland.

[4] Institute of Archaeology, Collegium Historicum, Adam Mickiewicz University, Poznan, Poland.

[5] Institute of Computing Sciences, Poznan University of Technology, Poznan, Poland.

[*] Correspondence and requests for materials should be addressed to:
Prof. Marek Figlerowicz
Institute of Bioorganic Chemistry
Polish Academy of Sciences
Noskowskiego 12/14
61-704 Poznan, Poland
email: marekf@ibch.poznan.pl

Materials and Methods

Comparative data for population genetic analyses

For comparative analyses of newly reported individuals living between Vistula and Bug Rivers in Iron Age (Mas-VBIA), we used prehistoric and present-day mitochondrial data from published sources.

Prehistoric comparative data

The mtDNA data of Kow-OVIA were compared with published prehistoric data from Europe and Western Eurasia. Those datasets were separated into groups based on cultural, chronological, and geographic features (Supplementary Table S7). Those include: Paleo-Mesolithic Hunter-Gatherers metapopulations: Central/North European Hunter-Gatherers (HGCN), Southwestern European Hunter-Gatherers (HGSW), East European Hunter-Gatherers (HGE). Early European Farmer groups (EEF) and the Neolithic cultures composed of the Starčevo Culture population (STA), Linearbandkeramik in Transdanubia (LBKT), Linearbandkeramik population from Central Europe (LBK), the Neolithic cultures representing temporal succession in Central European region: Rössen Culture (RSC), Schöningen Group (SCG), Baalberge Culture (BAC), Salzmünde Culture (SMC) and Bernburg Culture (BEC); Late Neolithic and Early Bronze Age cultures: Corded Ware Culture (CWC), Bell Beaker Culture (BBC) and Unetice Culture (UC); prehistoric populations from Southern Scandinavia: a Neolithic Hunter-Gatherer population from the Pitted Ware culture (PWC), contemporaneous with it individuals from the Funnel Beaker culture (TRB); individuals from Jutland Iron Age period (JIA); populations of Southwestern European origin: Cardial/Epicardial culture of the Iberian Penisula (CAR), Portuguese Neolithic population (NPO), Neolithic population from Basque Country and Navarre (NBQ), Iberian Chalcolithic El Mirador Cave individuals (MIR), individuals from Iberian Iron Age period (IIA). Additionally, we included population of the Treilles culture from Southeast France (TRE), Early/Middle Neolithic Paris Basin individuals from Gurgy 'Les Noisats' group (RRBP), Bronze Age Kurgan samples from South Siberia (BAS), Bronze Age population from Kazakhstan (BAK), sample set representing steppe herder population of Yamnaya culture (YAM), Iron Age Scythian samples (SCY), individuals from the Scytho-Siberian Pazyryk Culture (SSP), individuals living between Oder and Vistula Rivers in the Iron Age (Kow-OVIA). For references, see Supplementary Table S7.

Present-day comparative data

To unravel affinities of Mas-VBIA maternal gene pool to present-day populations, we compared our prehistoric data set to data from extant populations of Europe, Near East, Asia and Africa. The present-day data were pooled into 2 following comparative datasets, that were used as an input for different statistical methods:

I. Central European metapopulation (CEM, n=500), which represents the mtDNA variation in this region. Samples were randomly drawn from a pool of HVS-I sequences from Austria, the Czech Republic, Germany and Poland.
II. Dataset of 73 populations from Europe, Near East, Asia and Africa, used for haplogroup frequency Principal Component Analysis (PCA).

Analysis of genetic distances - substitution model and gamma value selection

Depending on the sample composition within each statistical test applied, we evaluated the most suitable substitution model and an associated gamma value separately for each analysis with the Akaike and Bayesian information criterion (AIC and BIC) in jModelTest 2.0 v.0.1.1. Genetic distances between CEPT populations were computed with Tamura & Nei substitution model and with an adjusted gamma-value of 0.312. Fst values between EPT populations were computed with Tamura & Nei substitution model and with an adjusted gamma-value of 0.3420.

AMOVA analyses were performed in Arlequin 3.5.1 using Tamura & Nei substitution model and a gamma value of 0.2650 (without CEM) or 0.312 (with CEM).

Supplementary Figures:

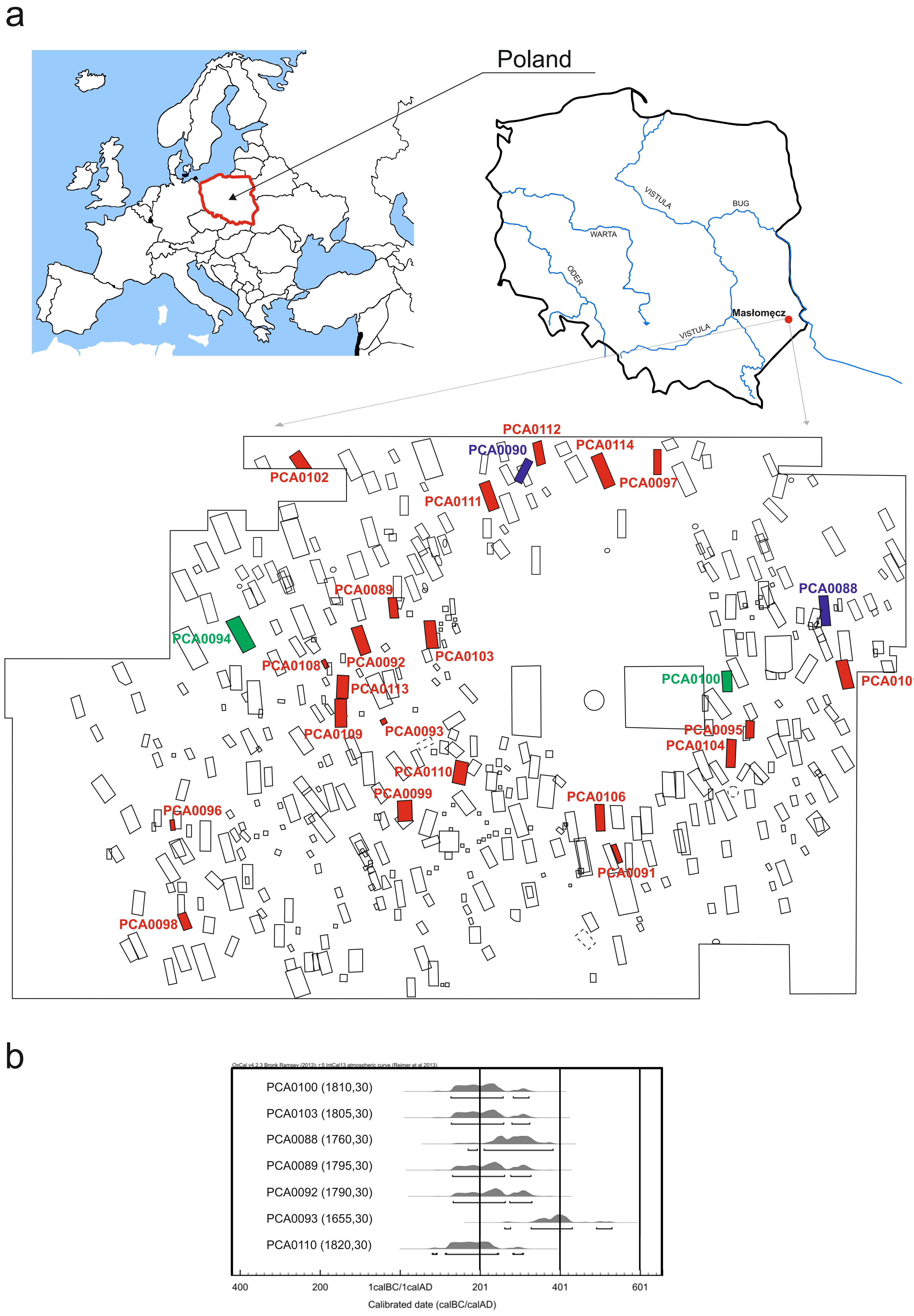

Figure S1

Location of Masłomęcz and a scheme of the Masłomęcz cemetery site 15, based on the Fig. 36 from the monograph by Andrzej Kokowski, Grupa masłomęcka: z badań nad przemianami kultury Gotów w młodszym okresie rzymskim, published in: Wydawnictwo Uniwersytetu Marii Curie-Skłodowskiej, Lublin 1995, and Fig. 1 from the monograph by Andrzej Kokowski, Cmentarzyska ludności grupy masłomęckiej, published in: Studia Antropologiczne V, Acta Universitatis Vratislaviensis No. 2050, Wrocław 1998, generated using Corel Draw ver. 12.0, with the author permission. Sampled graves are marked with a red color. Europe and Poland maps were downloaded from Wikimedia Commons (https://commons.wikimedia.org), under the free licence, and modified with Corel Draw ver. 12.0.

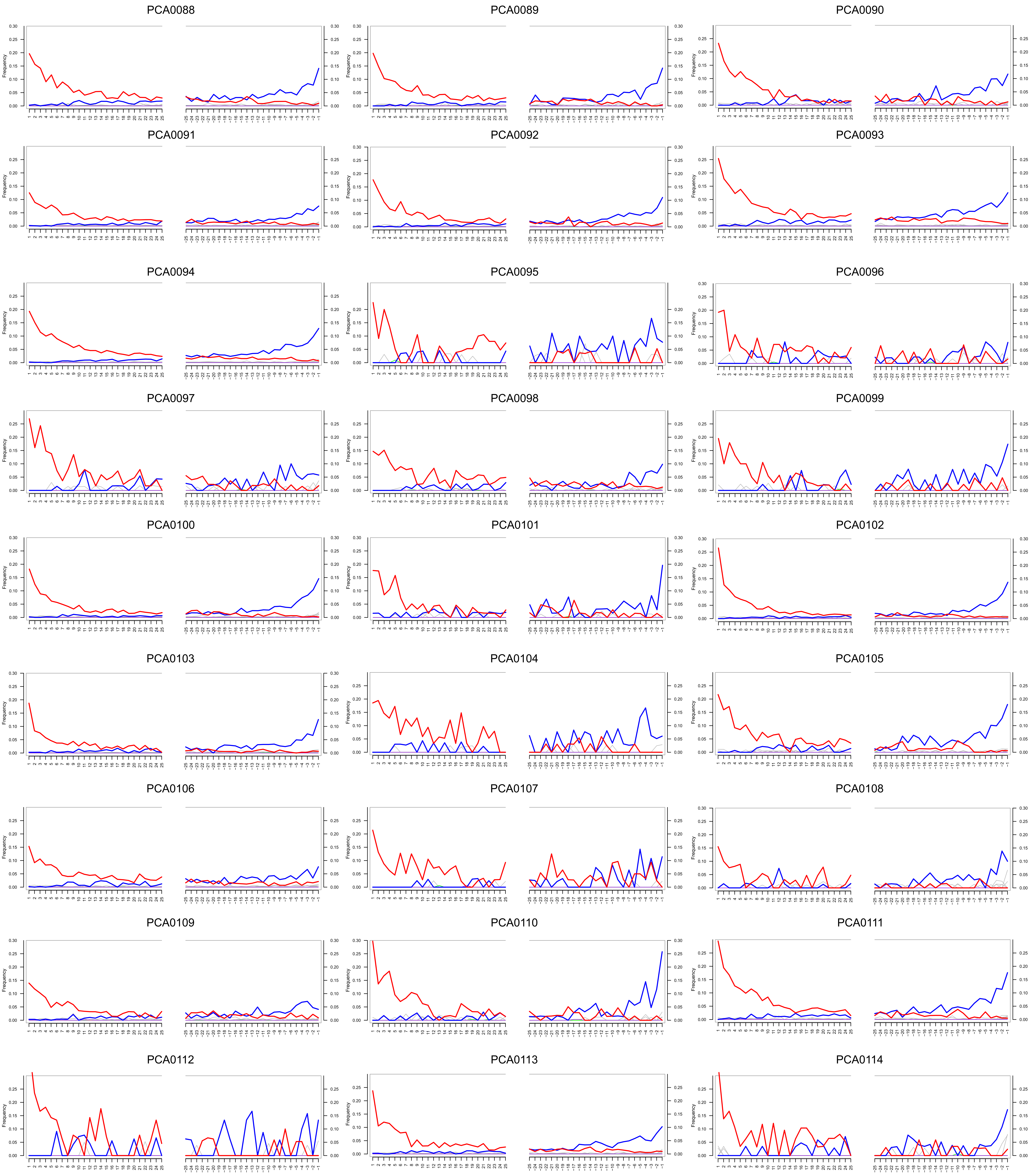

Figure S2

MapDamage 2.0 misincorporation plots showing damage patterns typical for aDNA.

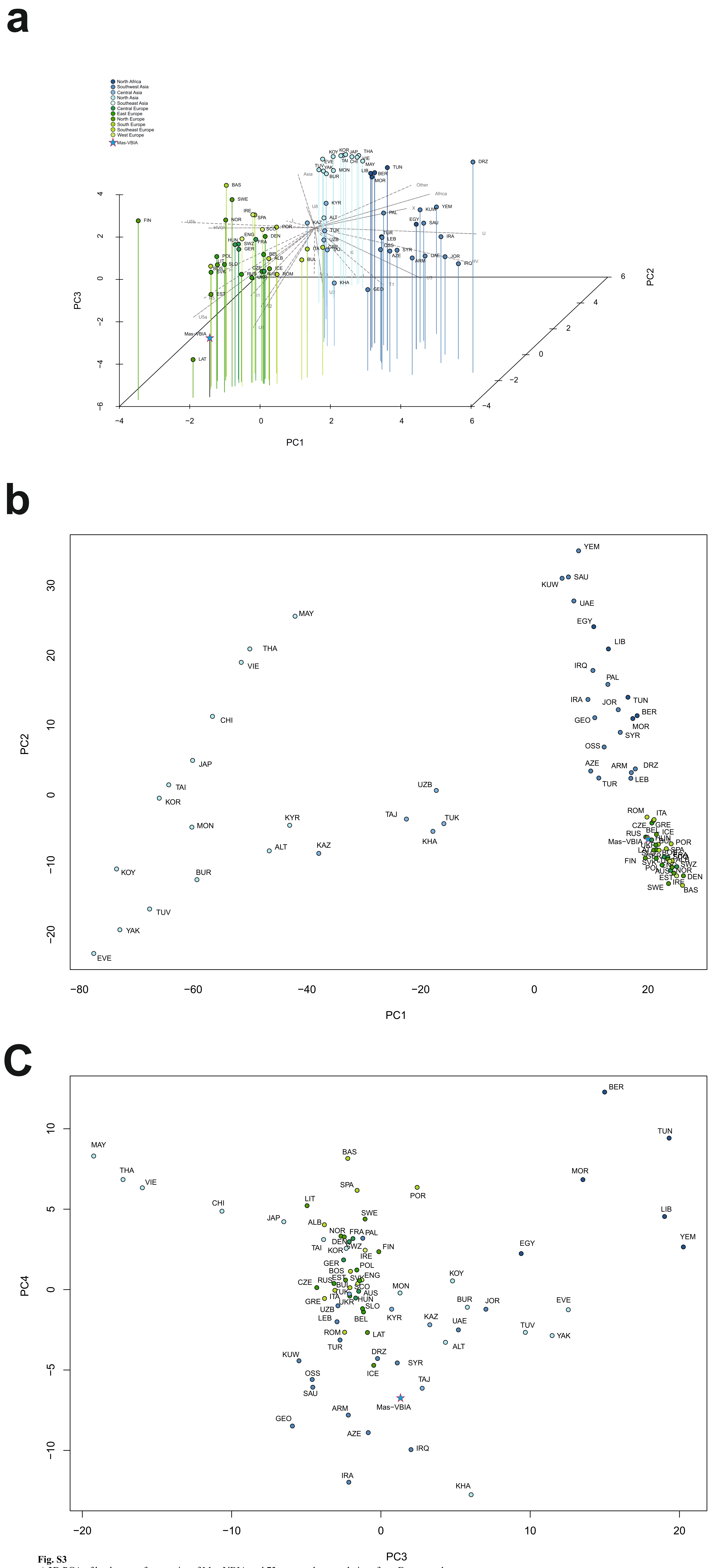

Fig. S3
a) 3D PCA of haplogroup frequencies of Mas-VBIA and 73 present day populations from Europe and Near East, b) PC1 and PC2 view, c) PC3 and PC4 view.
